# Pole position: How plant cells polarize along the axes

**DOI:** 10.1093/plcell/koab203

**Published:** 2021-08-02

**Authors:** João Jacob Ramalho, Victor Arnold Shivas Jones, Sumanth Mutte, Dolf Weijers

**Affiliations:** Laboratory of Biochemistry, Wageningen University, Stippeneng 4, 6703WE Wageningen, The Netherlands; Laboratory of Biochemistry, Wageningen University, Stippeneng 4, 6703WE Wageningen, The Netherlands; Laboratory of Biochemistry, Wageningen University, Stippeneng 4, 6703WE Wageningen, The Netherlands; Laboratory of Biochemistry, Wageningen University, Stippeneng 4, 6703WE Wageningen, The Netherlands

## Abstract

Having a sense of direction is a fundamental cellular trait that can determine cell shape, division orientation, or function, and ultimately the formation of a functional, multicellular body. Cells acquire and integrate directional information by establishing discrete subcellular domains along an axis with distinct molecular profiles, a process known as cell polarization. Insight into the principles and mechanisms underlying cell polarity has been propelled by decades of extensive research mostly in yeast and animal models. Our understanding of cell polarity establishment in plants, which lack most of the regulatory molecules identified in other eukaryotes, is more limited, but significant progress has been made in recent years. In this review, we explore how plant cells coordinately establish stable polarity axes aligned with the organ axes, highlighting similarities in the molecular logic used to polarize both plant and animal cells. We propose a classification system for plant cell polarity events and nomenclature guidelines. Finally, we provide a deep phylogenetic analysis of polar proteins and discuss the evolution of polarity machineries in plants.

## Teaser

This review discusses the principles, mechanisms, and evolution of plant cell polarity, an essential cellular feature for plant development and physiology that endows cells with a sense of direction.

## Introduction

Living systems display an astounding degree of spatial organization that is essential for their development and function. Key to this organization is the regulated asymmetric distribution of molecules and structures at the subcellular level, termed cell polarity. This ubiquitous phenomenon, which appears even in single-celled organisms, fueled the increase in morphological and functional complexity of life forms throughout evolution. The establishment of cell polarity provides cells with a coordinated system that can be interpreted at the cell or tissue level to spatially organize diverse cellular and developmental processes. In dividing cells, polarity can influence the orientation of the cell division plane or the segregation of fate determinants to generate daughter cells with different morphologies and developmental potential. These asymmetric cell divisions (ACDs) are responsible for cell type diversification in both prokaryotes and eukaryotes, and are essential for the development of multicellular organisms (Sunchu and Cabernard, 2020). Polarity is also crucial in nondividing cells: the spatial information provided by cell polarity can determine cellular morphology and function by instructing the positioning of organelles, cortical structures, and functionally relevant molecules, such as those involved in directional intercellular transport or pathogen perception (Klunder et al., 2017; Nakamura and Grebe, 2018). Similarly, migrating or expanding cells require a polarity axis that determines the localization and direction of migration or growth (Woodham and Machesky, 2014; Qin and Dong, 2015). Finally, the polarity of individual cells can be integrated within the plane of a tissue, known as planar cell polarity (PCP), to coordinate the patterning and positioning of structures at higher organizational scales (Butler and Wallingford, 2017).

Although multicellularity arose independently in plants and animals, in both lineages, cell polarity is vital in addressing problems of body organization and homeostasis. Unlike animals, however, plant cells are constrained by the presence of a rigid cellulosic cell wall. This prevents cell migration and morphogenic cell movements, which are the key features of animal development and allow animal cells to end up in locations distant from where they originated. Instead, cell lineage, position, and fate are inextricably linked in plants. Thus, precise selection of the cell division plane, often instructed by polarity, is critical for the generation and organization of different cell types into tissues and organs. In addition, polarity is often fundamental to the morphogenesis and physiology of individual cells.

Compared to animal cells, little is known about the molecular mechanisms underlying the establishment of plant cell polarity, perhaps due to the unique constraints posed by plant development. Beyond basic cellular processes, not much appears to be shared with the mechanisms of cell polarity in other lineages. However, insights gained from nonplant systems can be instructive regarding the general principles involved. In this review, we introduce a conceptual framework to guide further studies on plant cell polarity. We discuss recent developments regarding the mechanisms that establish and maintain polarity at both the cell and tissue level, provide a deep phylogenetic analysis of known regulators of cell polarity, and speculate on the evolution of polarity machineries within the plant kingdom.

## What is in a name? The flavors of plant cell polarity

The term “cell polarity” encompasses a diverse array of cases and processes in which proteins or other cellular components are unevenly distributed in the cell, but on a mechanistic level these phenomena do not necessarily form a coherent grouping. We suggest that they can usefully be classified into three categories: localized growth polarity, transient polarity, and axial polarity (Table 1). The major conceptual distinction between axial polarity and the two other types lies in their scale of applicability to cells within an organ. Localized growth polarity and transient polarity refer to the establishment of cellular asymmetry within an individual cell or small group of cells, distinct from the overall population in the organ. Examples of localized growth polarity include the tip growth of pollen tubes, root hairs, rhizoids, and protonema, in which growth is focused in a small domain of a single cell. An example of transient polarity comes from the stomatal lineage in *Arabidopsis thaliana*, in which patterns of polarization and subsequent oriented ACD occur during a limited period in a subsection of a leaf formed by descendants of a protodermal cell. In contrast, axial polarity is relevant for most cells in an organ independently of cell type and can be described as the basal coordinate system that guides the spatial organization of cellular processes along the major developmental body axes, such as the embryonic and post-embryonic root–shoot axis. Different types of polarity can be superimposed in the same cell, as demonstrated by the persistence of axial polarity markers in root hair-forming trichoblasts with disrupted localized-growth axis positioning (Pietra et al., 2013), or the restriction of a polar marker to a single lobe in pavement cells (discussed below; Mansfield et al., 2018), which show shape-defining alternating polar domains in lobes and indentations (Majda et al., 2017). In this review, we primarily focus on the principles and mechanisms underlying axial polarity.

**Table 1:** Key concepts in plant cell polarity

Term	Description
Cell polarity	Regulated asymmetric distribution of cellular components and structures along an axis
Polarity field	Individual cell polarities coordinated across tissues and organs
Polarity regulator	Contributes to polar domain formation and polar protein localization
Polarity effector or client	Uses pre-existing polar domains for localization
Axial polarity	Stable polarity axes aligned with the major body axes
Localized growth polarity	Polarity axis that determines the direction of and sustains (out)growth in specific cell types
Transient polarity	Transient polarity axis that determines morphological and/or fate asymmetry of cell division in specific cell types
Symmetry-breaking	Stimuli-induced or spontaneous initial local asymmetry that orients the polarity axis and initiates the polarization program
Reinforcement	Amplification of the initial asymmetry into stable polar domains via positive feedback and inhibitory mechanisms
Implementation	Recruitment of effectors to polar domains that drive polarization of cellular components, processes, and functions

## Coordination of cell polarities with axes and polarity fields

**Figure 1:**
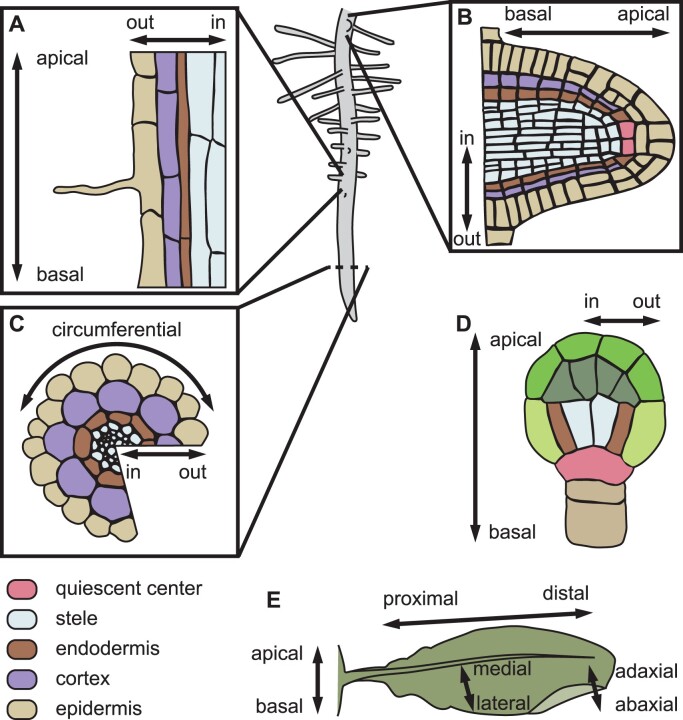
Major plant body axes. Diagrams indicate the major body axes in: (A) longitudinal root section; (B) transverse root section; (C) longitudinal section of a lateral root; (D) early embryo; (E) leaf. The identities of the root tissues in (A)–(C) are described in the color-coded legend.

Cell polarity provides a cell with control over its shape, division plane, or functions, but these are most meaningful when placed in the context of the organ the cell resides in. The Arabidopsis root has been a fruitful system for the study of cell polarization. The highly stereotyped cell divisions and the organized cell layers they give rise to make it straightforward to describe the polar distribution of cellular components with regard to the root’s major axes. The inner–outer axis extends from the center of the root to its surface: the inner domain of a cell is oriented toward the vasculature, and the outer domain toward the surface of the organ and the soil beyond (Figure 1, A–D). The apical–basal axis runs from the tip of the shoot toward the root, with cellular domains accordingly described as apical (shootward) or basal (rootward; Figure 1, A and B). The remaining two faces of the typical cuboid root cell, which face the neighboring cells of the same tissue layer perpendicular to the apical–basal axis, represent a distinct polarity that is neither inner–outer nor apical–basal. We suggest the term circumferential polarity for this rarely considered and poorly described orientation (Figure 1C). Further increasing the complexity of the system beyond cell faces, cell edges and corners also appear to form polarity domains with unique molecular composition (Elliott and Kirchhelle, 2019). Although only a handful of edge-localized proteins have been identified, these are involved in vesicular trafficking, microtubule nucleation and stability, and modification of cell wall properties, and likely have important functions in defining the biochemical and mechanical properties of cell edges.

**Figure 2:**
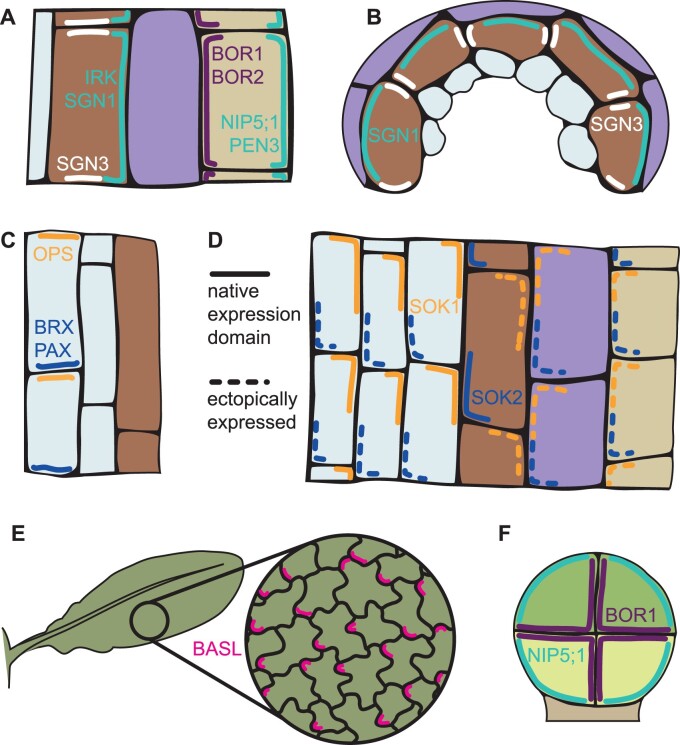
Proteins that polarize relative to the major plant body axes. A, In a longitudinal section of the root, NIP5;1 and PEN3 localize to the outer domain, while BOR1 and BOR2 localize to the inner domain. In the endodermis, IRK and SGN1 localize to the outer domain, and SGN3 is found in the apical and basal domains. B, In a transverse section of the root, SGN3 localizes to the circumferential domain of endodermal cells where neighboring cells of the same tissue layer meet. SGN1 localizes to the outer domain. C, In root protophloem cells, OPS localizes to the apical domain, while BRX and PAX localize to the basal domain. D, SOK1 is natively expressed in vascular cells, where it localizes to the apical outer corner. SOK2 is natively expressed in the endodermis, where it localizes to the basal inner corner. When ectopically expressed, SOK2 maintains the same polar localization in all cell layers. In contrast, although SOK1 remains apical in all cell layers, its distribution along the inner–outer axis is oriented toward the cortex-endodermis junction. Root tissues in (A)–(D) are color-coded as indicated in Figure 1. E, Ectopically expressed BASL throughout the leaf localizes to a single lobe of pavement cells aligned with the proximal–distal axis. F, Polar distribution of NIP5;1 to the outer domain and BOR1 to the inner domain in the eight-cell stage embryo.

A number of proteins are known to be polarized relative to the inner–outer axis of the root, many with functions related to the transport of small molecules. The directional transport of silicon from the soil to the stele in rice (*Oryza sativa*) is dependent on the polar distribution of importers (Lsi1) and exporters (Lsi2) to the outer and inner plasma membranes of outer root cell layers, respectively (Ma et al., 2006, 2007). Boron uptake in Arabidopsis is facilitated in a similar manner by the boron importer NOD26-LIKE INTRINSIC PROTEIN5;1 (NIP5;1), localized to the outer domain of root epidermal cells, and the exporters REQUIRES HIGH BORON1 (BOR1), and BOR2, localized to the inner domain of different root cell files (Alassimone et al., 2010; Takano et al., 2010; Miwa et al., 2013; Figure 2, A and F). Several proteins involved in pathogen defense, such as the ATP-BINDING CASSETTE G subfamily (ABCG) transporter proteins ABCG34/PDR6, ABCG36/PEN3, and ABCG37/PIS1, are also localized to the outer domain of root epidermal cells, facing toward invading pathogens (Strader and Bartel, 2009; Langowski et al., 2010; Khare et al., 2017). Finally, several developmental proteins display inner–outer polarity. The leucine-rich repeat receptor-like kinase (LRR-RLK) INFLORESCENCE AND ROOT APICES RECEPTOR KINASE (IRK) is localized to the outer domains of cells in the pericycle and endodermis, where it influences the plane of cell division (Campos et al., 2020; Figure 2A). The receptor-like cytoplasmic kinase SCHENGEN1 (SGN1) localizes to the outer faces of differentiating endodermal cells, spreading also to the circumferential, apical, and basal faces (Alassimone et al., 2016). Here, it positions CASPARIAN STRIP MEMBRANE DOMAIN PROTEIN (CASP) proteins and the future Casparian strip, where its activity overlaps with that of SGN3, which is located in a wide band along the apical, basal, and circumferential faces (Figure 2, A and B). SGN3 and CASP proteins may thus also represent a rare example of proteins polarized to the circumferential domain.

Among the proteins that display apical–basal axial polarity are the well-studied proteins involved in polar auxin transport (PAT). These are extensively reviewed elsewhere (Adamowski and Friml, 2015) and will not be covered in detail here. In brief, in the stele, the efflux transporters PIN-FORMED 1 (PIN1), PIN3, PIN4, and PIN7 are basally localized, directing auxin in the rootward direction. In an illustration that the polarity of a protein relative to body axes can be cell type specific, PIN3 and PIN7 lose their basal polarity in the columella, becoming apolar, while PIN2 is located basally in the cortex but apically in the epidermis and root cap (Müller et al., 1998; Gälweiler et al., 1998; Friml et al., 2002a, 2002b, 2003). Other proteins that polarize according to the apical–basal axis are found in developing protophloem sieve elements. BREVIS RADIX (BRX) and PROTEIN KINASE-ASSOCIATED WITH BRX (PAX) localize to the basal domain of these cells (Scacchi et al., 2009; Marhava et al., 2018), while OCTOPUS (OPS) is apically localized (Truernit et al., 2012; Figure 2C). The apical–basal polarity axis is also read out by BREAKING OF SYMMETRY IN THE STOMATAL LINEAGE (BASL), a polar scaffold protein important for stomatal lineage development. When this protein is ectopically expressed in the root, it localizes to the basal domain (Dong et al., 2009), presumably polarized by some common feature of this domain across the various cell types and layers of the root.

The existence of a widespread coordinate system in the root is also suggested by the polar localizations of the recently identified SOSEKI (SOK) proteins. This family is represented by five members in Arabidopsis, each of which localizes to specific corners and edges of cells (Yoshida et al., 2019). For example, SOK1 is localized to the outer apical corner and outer face of vascular cells, and SOK2 to the inner basal corner of endodermal cells. When ectopically expressed throughout the root, both SOK1 and SOK2 maintain their polarity relative to the apical–basal axis, even in cell types they are not normally present in (Figure 2D). This behavior suggests that, similar to ectopic BASL, they polarize according to an apical–basal polarity cue that is present in all the cells of the root, hinting at the existence of a global coordinate system. When a new lateral organ forms, such as the emergence of a lateral root, new axial polarity axes are established oriented relative to the direction of the organ’s growth rather than that of the parent axis, as evidenced by the analogous localization of polar markers (Benková et al., 2003; Mansfield et al., 2018; Yoshida et al., 2019).

In contrast to their consistent apical–basal localization, SOK1 and SOK2 differ in their polarization relative to the inner–outer axis when expressed in different tissue contexts. SOK2 is localized toward the inner domain, regardless of the tissue, whereas SOK1 always points toward the junction between the endodermis and cortex—that is, it is localized to the inner domain of cells on the outer side of this boundary (cortex and epidermis), and to the outer domain of cells on the inner side of it (stele and endodermis; Yoshida et al., 2019; Figure 2D). This indicates that the apical–basal and inner–outer polarity cues are genetically separable, and that polarity along the inner–outer axis may combine several independent cues. The behavior of other proteins also suggests that polarization can occur according to different cues or landmarks along the inner–outer axis. While the classical inner–outer markers BOR1 and NIP5;1 are always polarized toward the stele and soil, respectively (Alassimone et al., 2010), other proteins have been shown to polarize toward the cortex–endodermis junction, including SGN1 and IRK (Alassimone et al., 2016; Campos et al., 2020). This suggests the existence of some positional cue directing polarization toward the junction between these cell types. The nature of these cues, if and how both transmembrane and soluble factors are guided by the same cue, and the significance of the cortex–endodermis junction in lateralization and redirecting polarity are all interesting open questions.

Although the root is far better understood due to its ease of study, the aerial organs of plants surely possess proteins that display axial polarity. Stem tissues possess the same apical–basal and inner–outer axes as the root, according to which proteins could polarize (Gälweiler et al., 1998; Bennett et al., 2006; Yoshinari et al., 2016). In contrast, planar leaves have a different set of axes: proximal–distal (from the base of the leaf toward the tip), adaxial–abaxial (from the face nearest the shoot apex to the opposite face), and mediolateral (from the centerline of the leaf toward its edges; Figure 1E). There is growing evidence of axial cell polarity relative to the proximal–distal leaf axis. Various models of leaf development describe how cells can vary their anisotropic growth and division according to a proximal–distal axis. These models successfully recapitulate the shapes and, in some cases, the clonal lineages or mutant phenotypes observed in the morphogenesis of planar leaves (Kuchen et al., 2012; Richardson et al., 2016), petals (Rebocho et al., 2017), and the highly curved traps of *Utricularia* (bladderworts; Lee et al., 2019; Whitewoods et al., 2020), suggesting the existence of a polarity field in planar lateral organs throughout their development. Consistent with this hypothesis, it has long been known that the polarity of trichomes is coordinated across the entire Arabidopsis leaf, with the asymmetrically branched hairs aligned according to the proximal–distal axis of the leaf (Hülskamp et al., 1994; Bouyer et al., 2001). In other species, similar morphological polarization relative to the proximal–distal leaf axis has also been observed, such as that of trichomes in barley (Richardson et al., 2016) and quadrifid glands in the traps of *Utricularia* (Lee et al., 2019).

As with the root, ectopic expression of BASL has been useful in reading out polarity in the leaf. The existence of a polarity field of the kind proposed in models has been demonstrated at the molecular level by Mansfield et al. (2018). Although BASL is usually only expressed in stomatal lineage cells, in the ectopic environment of the jigsaw puzzle-shaped pavement cells, it localizes to a single lobe out of multiple seemingly identical lobes. The use of a genetic background that lacks stomata removed the confounding factor of spiral patterns of BASL polarity in stomatal domains, and revealed that the polarity of each pavement cell is strongly aligned to the proximal–distal axis of the leaf (Figure 2E). The alignment is strongest along the midrib, but diverges toward the lamina during later growth stages; this pattern is similar to the observed growth directions of cells during leaf development, and to the hypothetical polarity field proposed to account for them by modeling (Kuchen et al., 2012). Strikingly, the polarity field that ectopic BASL responds to exists throughout leaf development and persists in interphase pavement cells, indicating that the coordination of cell polarities is not a transient phase during morphogenesis but rather is maintained even in the mature leaf. Thus, although no proteins have yet been discovered that natively display axial polar localization in the leaf, ectopically expressed BASL provides a readout demonstrating that these cells are axially polarized.

## Mechanisms for the establishment of axial cell polarity

**Figure 3:**
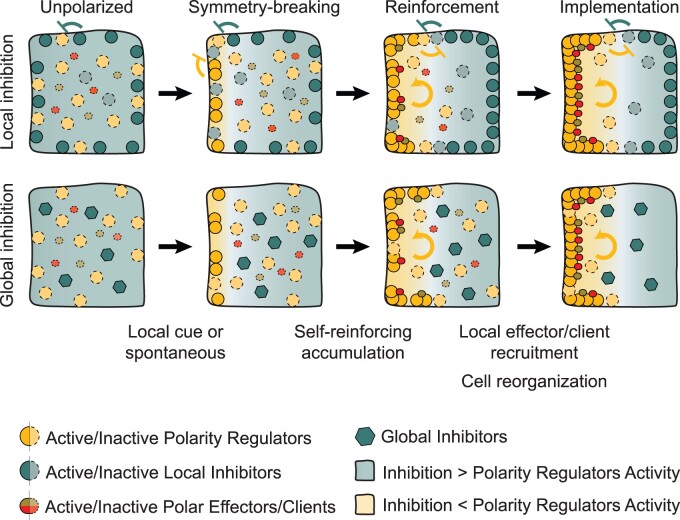
Mechanistic principles of cell polarity establishment. Schematic representation of two common mechanisms of cell polarity establishment in eukaryotes. Symbols represent one or multiple molecular entities. Curved arrows represent self-reinforcing interactions, and flat-ended arrows represent inhibitory interactions.

A polarized cell can be identified based on morphological asymmetries, such as overall cell shape and biased localization of organelles, or by the asymmetric distribution of molecules such as lipids, proteins, and RNA. The existence of several axially polarized proteins has been known for decades, but our mechanistic understanding of how plant cells establish these polar domains is rudimentary compared to what is known in other eukaryotes or localized growth polarity in plants. Moreover, most known polar proteins in plants appear to be “clients” or “effectors” of the polarity system rather than regulators. We operationally define a regulator as a protein that influences the polar localization of other proteins, and clients as those that use existing polarity information for their targeting, without themselves controlling aspects of the polarity system (Table 1). Clearly, the nonlinear and nondeterministic nature of plant development and molecular signaling obscures the absolute binning of proteins as one type or the other, but within a given context, the regulator/client definition may help to clarify functional relationships. Multidisciplinary research across model organisms has revealed that the establishment of cell polarity is a self-organized process driven by a complex network of biochemical and biomechanical interactions that transform a local heterogeneity or noise into a stable cell-wide asymmetry (Li and Bowerman, 2010; Goehring and Grill, 2013). This process is hierarchical and can be broken down into three major steps: symmetry-breaking, reinforcement (or amplification) of the asymmetry, and implementation of this asymmetry to affect other cellular processes (Figure 3 and Table 1). In the following sections, we will explore each of these steps and how they apply to axial cell polarity in plants.

### Symmetry breaking

As a first step in polarization, cells must undergo a symmetry-breaking event. These events are local and often transient, triggering a subsequent cellular program that reinforces the initial asymmetry in order to polarize the entire cell. Diverse cues have been described that can drive symmetry-breaking in biological systems. These can be extrinsic to the system, such as light (Kropf et al., 1999), mechanical stress (Hikita et al., 2018), diffusible molecules (McDonald et al., 2010), cell–cell or cell–matrix contacts (Yu et al., 2005), and the sperm entry site in egg cells (Hable and Kropf, 2000). These cues can also be cell-intrinsic, such as inherited landmarks from the system’s past, for example a remnant of a prior cytokinetic event (Park et al., 1999; Lam et al., 2006). Spatial cues are not always required, but can serve to orient or increase the efficiency and robustness of polarization in systems that are already poised to polarize (Wedlich-Soldner and Li, 2003). In these spontaneously polarizing systems, symmetry-breaking depends on stochastic fluctuations and eventual accumulation of polarity regulators at the cell cortex, which establish, at least initially, a randomly oriented axis, as recently exemplified by polarization of BASL in isolated protoplasts (Chan et al., 2020).

Unambiguous identification of a symmetry-breaking cue is rarely a trivial undertaking due to their often discrete spatiotemporal nature and their function in activating asymmetry amplification, which complicate the task of disentangling concurrently acting mechanisms. In most contexts in land plants, there is the added challenge that cells are rigidly embedded in a tissue that can provide spatial cues. Thus, it may be unsurprising that the symmetry-breaking mechanisms that establish the different axial polar domains remain elusive. In Arabidopsis, polarity along the apical–basal axis is already evident in the egg cell prior to fertilization by the asymmetric distribution of organelles and longitudinal orientation of microtubules (MTs; Mansfield and Briarty, 1991; Ueda et al., 2011). These hallmarks of polarity are transiently lost upon fertilization but reemerge in the zygote, which elongates along the apical–basal axis prior to the first transverse cell division (Ueda et al., 2011; Kimata et al., 2016). The zygotic division is markedly asymmetric, generating daughter cells with different morphologies and fates. Subsequent development of the smaller apical daughter, which forms most of the proembryo, involves successive changes in division plane orientation in a series of highly predictable symmetric and asymmetric divisions (ten Hove et al., 2015).

Despite significant progress in uncovering molecules involved in zygotic elongation as well as fate and morphological division asymmetry, it is currently unclear how the apical–basal axis arises. Fertilization causes the egg cell to lose its former high degree of polarization (Ueda et al., 2011; Kimata et al., 2016), and elongation and subsequent division asymmetry require zygotic genome activation (Kao and Nodine, 2019; Zhao et al., 2019). However, the existence of a persistent polarizing signal from the egg cell that guides axis establishment in the zygote cannot be excluded. In some animals and fucoid brown algae, the sperm entry site can provide an orienting cue for polarity establishment (Hable and Kropf, 2000; Piotrowska and Zernicka-Goetz, 2001). However, experiments with isolated in vitro-fertilized rice zygotes showed no correlation between the sperm entry site and the orientation of the first cell division (Nakajima et al., 2010). Given that the Arabidopsis ovule and embryo sac are themselves polarized structures (Mansfield and Briarty, 1991), that the zygote is attached to the maternal tissue at a specific position, and the both zygote elongation and subsequent divisions follow a robust and reproducible orientation relative to the embryo sac (Lukowitz et al., 2004; Yoshida et al., 2014), an alternative explanation would be that a signal from the surrounding tissues determines the apical–basal axis in the zygote. Whether this is the case, and what the signal might be, are still conjectures. Maternally provided auxin could act as a putative symmetry-breaking signal (Robert et al., 2018), but with the exception of the guanine nucleotide exchange factor for ADP-ribosylation factor (ARF-GEF) GNOM, whose role in the zygote is not fully understood (Steinmann, 1999; Geldner et al., 2003), defects in auxin-related mutants are only detected after the formation of the apical–basal axis (Möller and Weijers, 2009).

The origin of the inner–outer axis is equally mysterious. The ectopic expression of the inner–outer domain boron transporter pair BOR1 and NIP5;1 using an embryo-specific promoter revealed that inner and outer membranes already have a distinct molecular composition as early as the four-cell stage, before multiple tissue layers have developed (Alassimone et al., 2010; Liao and Weijers, 2018). The establishment of this polarity axis precedes any known physiological need for nutrient exchange and the secretion of the protective cuticle by peripheral cells (Rodkiewicz et al., 1994; Alassimone et al., 2010), arguing for a function for inner–outer polarity during early development beyond localized transport. Although specific sorting and trafficking pathways have been implicated in maintaining the polarized distribution of inner–outer cargoes during post-embryonic development (e.g. Langowski et al., 2010, 2016; Mao et al., 2016; Wang et al., 2017a; Yoshinari et al., 2019), to our knowledge, no mutants that perturb the establishment of the inner–outer polarity axis itself have been identified. Disruption of auxin signaling leads to aberrant orientation of cell division planes beginning at the two-cell stage (Hamann et al., 1999; Yoshida et al., 2014), suggesting that auxin could be involved in inner–outer axis formation or its integration with cell division orientation. Still, alternative mechanisms exploited by other eukaryotes remain mostly unexplored in plants. The presence of a cytokinetic landmark (Lujan et al., 2017), recognition of cell–cell or cell–free surfaces (Nance and Priess, 2002), and mechanical or biochemical heterogeneities between internal and external membranes (Prager-Khoutorsky et al., 2011; Nagata et al., 2021) could all serve as initial cues for inner–outer axis establishment that are propagated in subsequent divisions.

It is not clear whether newly formed cells after early embryogenesis ever require symmetry-breaking from a completely unpolarized state, as a polarized state could be inherited from the mother cell. For instance, lateral cargoes such as BOR1 retain their polarized localization in transversely dividing root cells (Yoshinari et al., 2016), despite the transient rerouting of cellular trafficking pathways toward the cell plate (Geldner et al., 2001; Dhonukshe et al., 2006; Men et al., 2008; Mravec et al., 2011; Richter et al., 2014; Glanc et al., 2018). Persistent polar localization is likewise seen for apical–basal cargoes in root cells undergoing transverse divisions. In the apical daughter cell, the redirection of trafficking toward the cell plate causes PIN2 to transiently accumulate in the basal domain, but the prior apical PIN2 domain is also maintained, whereas the NPH3-like protein NAKED PINS IN YUC MUTANTS 5 (NPY5), also known as MAB4/ENP/NPY1-LIKE 1 (MEL1), remains exclusively localized to the apical membrane of the apical daughter during division (Glanc et al., 2018). Thus, while newly formed cells need to redefine the polar identity of at least one membrane domain and reroute trafficking machineries, symmetry-breaking per se might be unnecessary, as a partially polarized state is inherited from the mother cell.

### Reinforcement

Reinforcement (conceptually similar to amplification) mechanisms are the core components of polarity establishment that can turn stochastic fluctuations or stimulus-induced local asymmetries into stable cell-wide asymmetries. Theoretical and experimental evidence gathered for over half a century has demonstrated that reinforcement in biological systems is heavily reliant on positive feedback loops that arise from interactions between regulatory proteins, referred to as polarity regulators, and structural components such as the cytoskeleton and the plasma membrane (Freisinger et al., 2013; Goehring and Grill, 2013;Thompson, 2013). These positive feedback loops translate into a self-reinforcing accumulation of polarity regulators, either at a specific domain when defined by a spatial cue or stochastically, but always in close association with the plasma membrane. Although the localization of polarity regulators at the cell cortex seems to be required for stable polarization, cytoplasmic and nonpolar regulators are often involved in this process. Notably, most polarity regulators described so far do not have any transmembrane domains and are instead peripherally associated with the plasma membrane. The direct association of soluble polarity regulators with the membrane can be mediated by specific lipid-binding domains or post-translational acylation (e.g. palmitoylation). Both are reversible, allowing for plasticity in the establishment of polar domains, and such reversibility is often the basis for inhibitory mechanisms that stabilize the polar domains (discussed below).

Numerous molecular interactions are able to generate the positive feedback loops required for self-reinforcing accumulation at the membrane. Examples include cortical recruitment and stabilization of polarity regulators by complex formation (Hong et al., 2001; Kozubowski et al., 2008), recruitment of molecules that alter the lipid composition of the membrane to favor membrane association (Weiner et al., 2002; Marhava et al., 2020), or the simple and often employed strategy of oligomerization (Dickinson et al., 2017; Gamblin et al., 2018). Self-reinforcement, however, is not sufficient to generate a stably polarized cell, as unrestricted accumulation of polarity regulators would eventually spread throughout the whole cell cortex. To ensure the spatial restriction of the polar domain and the singularity of the polarity axis, reinforcement mechanisms must therefore include features that counteract the self-sustained accumulation of polarity regulators. Such features include rapidly diffusing factors (e.g. GTPase-activating proteins or GDP-dissociation inhibitors) in Cdc42-driven polarization; Freisinger et al., 2013; Woods et al., 2016), mechanical forces (e.g. membrane tension; Houk et al., 2012), or even limiting pools of polarity regulators (Goehring et al., 2011), which can all function as global inhibitors. In addition, membrane-associated factors can act as local inhibitors preventing the spread of the polarity regulators across the membrane (Fletcher et al., 2012).

Stable polarization requires tight regulation of both concentration and activity of polarizing and inhibitory molecules. We will draw on anterior–posterior polarization of the nematode *Caenorhabditis elegans* one-cell embryo as an illustrative example of roles for both in reinforcement. Polarization of this system is centered on the conserved mutual antagonism between the anterior and posterior PAR modules (aPAR and pPAR, respectively), resulting in an asymmetric positioning of the division plane and fate determinants (Lang and Munro, 2017). The formation of the aPAR module depends on the scaffold protein PAR-3, which recruits the scaffold PAR-6 together with the aPKC kinase (Beers and Kemphues, 2006; Li et al., 2010). Anterior enrichment of the aPAR module is redundantly promoted by distinct positive feedback loops, among which oligomerization of PAR-3 is of particular relevance. Oligomerization of PAR-3, which directly interacts with membrane lipids (Krahn et al., 2010; Claret et al., 2014), increases its avidity for membrane association and interaction with PAR-6/aPKC and allows PAR-3 to be efficiently segregated to the anterior pole by aPAR-dependent cortical flows (Dickinson et al., 2017; Rodriguez et al., 2017; Wang et al., 2017b). aPAR also stimulates local enrichment of the lipid phosphatidylinositol-4,5-biphosphate (PI(4,5)P_2_), which in turn promotes aPAR polarity (Scholze et al., 2018), possibly via an interaction between PAR-3 and PI(4,5)P_2_ (Krahn et al., 2010; Claret et al., 2014). pPAR proteins PAR-1, PAR-2, and Lgl all interact directly with membrane lipids and self-reinforce via positive feedback (Moravcevic et al., 2010; Motegi et al., 2011; Dong et al., 2015). As PAR-3, PAR-2, and Lgl form oligomers, which for PAR-2 was shown to increase membrane residence time (Arata et al., 2016; Strand et al., 1994). Critically, membrane association of aPAR and pPAR modules is negatively regulated by PAR-1- and aPKC-dependent phosphorylation, respectively (Hao et al., 2006; Hoege et al., 2010; Motegi et al., 2011). When coupled to self-recruitment and limiting pools of PAR proteins, this kinase-driven mutual antagonism in cortical localization is the critical step that stabilizes the boundary between the opposing polar domains.

Sequencing of numerous plant genomes and transcriptomes has revealed that the known regulators of polarity in yeast and animals are not present in plants (Kania et al., 2014), and that plants must have evolved their own mechanisms to generate stable cellular asymmetries. A notable exception is RHO OF PLANTS (ROP) signaling in the polarization of different cells undergoing localized growth, which functions in an analogous manner to the distantly related small GTPase Cdc42, a master regulator of polarity in other eukaryotes (Yang, 2008; Scholz et al., 2020). Similar to other systems, ROP activity is tightly controlled by both activating (Gu et al., 2006; Denninger et al., 2019) and inhibitory (Hwang et al., 2010) factors, and it also involves local restriction by scaffold proteins (Kulich et al., 2020). Beyond this significant progress in uncovering the molecular mechanisms of localized growth, examples of reinforcement mechanisms in axial polarization are surprisingly scarce and limited to specific tissues and lineages. Although the conserved regulators of axial polarity in plants remain mysterious, research for the last few years has started to reveal some striking parallels in the molecular circuitry used to reinforce and stabilize asymmetries in the different kingdoms.

Mutant screens for altered PIN, BOR1, and CASP localization have identified several regulators of membrane biogenesis and trafficking (Feraru et al., 2011; Lin et al., 2012; Uehara et al., 2014; Alassimone et al., 2016; Rakusová et al., 2019; Zhang et al., 2020). However, whether such proteins should be considered polarity regulators is debatable, since while they affect the localization of other proteins, they do not appear to play a role in defining the identity of the domain itself. The characterization of polarity regulators in plants has thus been mostly limited to transient polarity during stomatal lineage development in Arabidopsis. This lineage starts with a markedly asymmetric division of meristemoid mother cells that produces daughter cells with different sizes and fates: a smaller meristemoid and a larger stomatal lineage ground cell (SLGC), which can undergo additional ACDs before differentiating into a pavement cell (Bergmann and Sack, 2007). It is well established that both daughter cell size and fate asymmetry depend on the prior formation of a crescent-shaped polar domain that is inherited by the larger SLGC (Dong et al., 2009). Reminiscent of the situation in animals, formation of the polar crescent and proper division asymmetry require cortical localization and activity of scaffold proteins such as BASL, POLAR, and members of the BRX family (BRXf; Dong et al., 2009; Pillitteri et al., 2011; Gudesblat et al., 2012; Bringmann and Bergmann, 2017; Rowe et al., 2019).

None of these proteins contain transmembrane domains, but mechanisms for cortical localization and self-reinforcement are now starting to emerge. Both BASL and BRXf contain putative palmitoylation sites that could confer membrane association. Indeed, mutation of the putative sites severely compromises cortical localization and function of BRXf proteins (Rowe et al., 2019). Similar experiments do not show an impact of palmitoylation on cortical localization of BASL (Zhang et al., 2016a), which instead appears to be directly recruited by BRXf (Rowe et al., 2019). This is consistent with the observation that BRXL2 polarizes before the formation of the BASL crescent (Gong et al., 2021). However, it is likely that BASL helps to focus the distribution of BRXf, as BRX and BRXL2 were reported to be more uniformly distributed across the membrane in *basl* mutants (Rowe et al., 2019). Interestingly, the essential function of the BASL polarity module in cell fate asymmetry is mediated by scaffolding components of fate determinant signaling pathways at the polar crescent, which in turn promote BASL polarity (Pillitteri et al., 2011; Zhang et al., 2015; Houbaert et al., 2018). Specifically, cortical localization and stability of BASL are promoted by direct regulatory interactions with mitogen-activated protein kinase (MAPK) pathway components such as the MAPK kinase kinase YODA, as well as MAPK3 and MAPK6, at the polar crescent (Zhang et al., 2015, 2016b). The cortical localization of BASL is also promoted by kinases of the GLYCOGEN SYNTHASE KINASE3 (GSK3) family such as BRASSINOSTEROID INSENSITIVE2 (BIN2), which localize to the crescent via POLAR to regulate cell fate (Houbaert et al., 2018).

Despite the significant progress in identifying components and their function in this system, there are still exciting questions ahead. Biochemical and mechanical stimuli have been proposed as cues that define crescent positioning (Dong et al., 2009; Bringmann and Bergmann, 2017), but their relative contributions and whether other positional cues exist is unclear. Further research is also necessary to understand the relationship between BASL and POLAR/BIN2, as pairwise interactions between BASL and POLAR or phosphorylation of BASL by BIN2 have not been demonstrated in vivo. Of note, inhibitors of the BRXf–BASL module that act to delimit the crescent have not yet been identified.

Another recently described system that shares some characteristics with animal reinforcement mechanisms operates during protophloem sieve element differentiation in Arabidopsis. In protophloem cells, the AGC-family member PROTEIN KINASE-ASSOCIATED WITH BRX (PAX) localizes to the basal membrane where it recruits the putative scaffolding protein BREVIS RADIX (BRX) (Marhava et al., 2018, 2020). In turn, the PAX–BRX complex promotes the recruitment of the phosphatidylinositol-4-phosphate (PI4P) 5-kinases PIP5K1 and PIP5K2, which convert PI4P into PI(4,5)P_2_, likely via a BRX–PIP5K interaction (Marhava et al., 2020). As seen for animal PAR-3, membrane association of PAX depends on PI(4,5)P_2_ (Barbosa et al., 2016; Platre et al., 2018; Marhava et al., 2020). Thus recruitment of PIP5K results in the self-reinforcing accumulation of PAX–BRX–PIP5Ks at the plasma membrane. It is proposed that this polarity module induces PI(4,5)P_2_-dependent endocytosis of PIN1 mostly at the center of the basal domain, leading to a distinctive “donut-like” PIN1 distribution required for sieve element differentiation, which is complementary to the “muffin-like” or super-polar distribution of PAX–BRX–PIP5Ks (Marhava et al., 2020). Notwithstanding mechanistic similarities with mammalian polarity reinforcement, the PAX–BRX–PIP5K module is unlikely to be a core regulator of cell polarity. PIN1 still localizes to the basal membrane in *pax*, *brx*, or *pip5k1/2* mutants, promotion of endocytosis was not observed for other integral membrane proteins (Marhava et al., 2020), and PAX–BRX polarity is maintained in *pip5k1/2* mutants, suggesting that the initial basal targeting of PAX is PIP5K-independent. Further analysis of additional polarity markers should clarify whether this module can confer basal membrane identity in developing sieve elements or simply act as a client of a pre-existing polarity axis to fine-tune PAT.

Lastly, SOK proteins may also play an important role in polarity reinforcement. While their function or relevance for polarity establishment remains obscure since no mutant phenotypes have yet been reported in *sok* mutants in Arabidopsis (Yoshida et al., 2019), a framework for how SOKs localize to the cell cortex is starting to surface. SOK proteins appear to be ubiquitous in land plants and primarily localize to specific cell edges in Arabidopsis, as well as in bryophytes such as the liverwort *Marchantia polymorpha* and the moss *Physcomitrium patens* (Yoshida et al., 2019; van Dop et al., 2020). Among the conserved features in all analyzed SOK sequences is the presence of a “CG” motif, which constitutes a putative palmitoylation site. Mutations within this motif abolish membrane association of Arabidopsis SOK1 and SOK5 (van Dop et al., 2020), suggesting that the membrane targeting of SOK proteins may depend on palmitoylation at this site. Notably, experiments with SOK chimaeras identified a discrete region that determines the preference of SOKs for specific polar domains (Yoshida et al., 2019). Understanding how this region influences polar domain selection might provide valuable insight into the molecular code underlying the axial coordinate system and how it is read by soluble factors.

**Figure 4:**
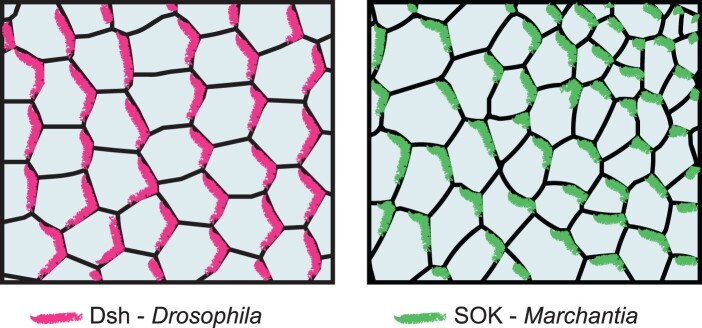
DIX domain-containing proteins mark polarity fields in animals and plants. Schematic representation of the polar localization of Dsh along the proximal–distal axis in the *Drosophila* wing epithelium (left), and MpSOK oriented toward the meristematic cell in the notch region of a *Marchantia* gemma (right). The localization patterns shown reflect the in vivo localizations of both proteins, as observed by confocal microscopy.

In stark contrast to most known plant polar proteins, the localization of Arabidopsis SOKs appears robust to numerous perturbations, including disruption of trafficking systems and the cytoskeleton, but is dependent on cell-wall integrity (Yoshida et al., 2019). Interestingly, the polar localization of the single *M. polymorpha* SOK in gemmae (van Dop et al., 2020) shows a striking resemblance to that of the conserved PCP component Dishevelled (Dsh) in the fruit fly (*Drosophila melanogaster*) wing (Figure 4;  Axelrod, 2001). The similarities with Dsh are not limited to localization pattern, as both proteins contain a structurally and functionally homologous N-terminal DIX domain. Despite high-sequence divergence, the structures of Arabidopsis SOK4 and human DVL2 DIX domains are virtually identical, and swapping of SOK and DVL2 DIX domains produce functional chimeras in cross-kingdom complementation assays (van Dop et al., 2020). Just like its animal counterpart, the DIX domain mediates concentration-dependent oligomerization of SOK proteins and is essential for their polar localization and function (van Dop et al., 2020). Arabidopsis SOK1 mutants that are unable to polymerize localize uniformly to the cell cortex (Yoshida et al., 2019; van Dop et al., 2020), suggesting an oligomerization-dependent inhibition of lateral diffusion that might be contingent on increased membrane-binding avidity, as seen for PAR-3 (Dickinson et al., 2017). Further extending the mechanistic similarities between SOKs and Dsh, as well as PAR-3, oligomerization of SOK proteins likely functions to increase the avidity for interaction partners (Schwarz-Romond et al., 2007; Dickinson et al., 2017), as supported by the polymerization-dependent recruitment of ANGUSTIFOLIA (AN) by Arabidopsis SOK1 (van Dop et al., 2020). These characteristics make it tempting to speculate that SOKs could be part of a conserved self-reinforcing mechanism that defines local membrane identity in a coordinated manner between cells.

In conclusion, despite molecular divergence, it appears that similar mechanistic principles underlie the origin of self-organized subcellular patterns in both plant and animal cells. Examples include the exploitation of reversible membrane association, oligomerization, modulation of the lipid microenvironment, as well as central roles of scaffolding in the assembly of polarity regulatory complexes and of phosphorylation in driving self-reinforcing loops. In addition to helping us understand the basic circuitry behind cellular pattern formation, these similarities reveal the usefulness of integrating the knowledge from the more developed animal cell polarity field to guide future research into the establishment of plant cell polarity.

### Implementation

The accumulation of polarity regulators at the cell cortex triggers a polarity program that segregates molecules and cellular processes according to the new axis, effectively imparting distinct characteristics and functions to each domain. Implementation of polarity programs commonly involves altering the molecular composition of the plasma membrane and cell cortex (and cell wall in plants), concomitantly with the spatial reorganization of vesicular trafficking, cytoskeleton, and organelles.

A common theme appears to be the feedback between cellular reorganization and domain amplification and stability. The above-described mutual self-reinforcement of phosphoinositides and polarity regulators in animals illustrates how altering membrane composition both regulates and implements a polarity program, as these lipids have conserved roles in trafficking, membrane organization, and molecular composition of the cell cortex (Hammond and Hong, 2018). Despite the asymmetric localization of some phosphoinositide species and their involvement in self-reinforcing polar modules in axially polarized plant cells (Tejos et al., 2014; Marhava et al., 2020), their contribution to the polar domain formation has only been proposed during localized growth (Stenzel et al., 2008; Stanislas et al., 2015). Similar synergistic interactions have been described for both actin and MT networks and associated vesicular trafficking systems in other eukaryotes (Siegrist and Doe, 2007; Orlando and Guo, 2009) as well as localized growth polarity in plants (Hwang et al., 2008; Yanagisawa et al., 2018; Ge et al., 2020). For example, the establishment of the intercalating polar domains driving localized growth in puzzle-shaped Arabidopsis pavement cells was recently shown to rely on a positive feedback between reorganization of cortical MTs and stabilization of clusters of polarity regulators. The formation of sterol- and TRANSMEMBRANE KINASE1-enriched clusters downstream of auxin induces local activation of ROP6 and subsequent reorganization of cortical MTs, which are in turn required for stabilization and coalescence of the proteo-lipid clusters (Pan et al., 2020).

Experimental evidence for similar feedback mechanisms during the establishment of axial polar domains, in addition to the critical role of these processes in domain specialization, is lacking. This knowledge gap is partly explained by the lack of known bona fide regulators of axial polarity, as well as the scarce availability and use of distinct reporters to probe domain identity, which is generally limited to factors that do not necessarily reflect overall polarity (e.g. PINs or other transmembrane proteins). Demonstrating the latter point, after division of epidermal cells, the peripherally membrane-associated NPY5 protein polarizes to the newly formed apical membrane much more rapidly than PIN2 (Glanc et al., 2018), a commonly used marker for polarity that is dependent on rerouting of trafficking from the cell plate. Although the effects of trafficking disruption on NPY5 polarity were not analyzed, the faster polarization of a soluble protein strongly suggests that, at least initially, re-establishment of apical identity occurs independently of trafficking.

The dramatic subcellular changes downstream of polarity are mediated by the localized recruitment of effectors to the cell cortex. The repertoire of polarity effectors varies based on the specific functions of each cell and is dynamically altered throughout the development and in response to environmental conditions. The number of known polar proteins in plants has been steadily increasing over the last few decades. For some, functions are understood in some detail. These include the previously mentioned function of stomatal polarity regulators in determining cell fate through spatial restriction of signaling components (Zhang et al., 2015; Houbaert et al., 2018), and the positioning and formation of the extracellular diffusion barrier known as the Casparian strip in endodermal cells (Roppolo et al., 2011; Lee et al., 2013; Pfister et al., 2014; Alassimone et al., 2016; Kalmbach et al., 2017; Doblas et al., 2017; Nakayama et al., 2017; Fujita et al., 2020). However, for most polar proteins, insights into the mechanisms that define their subcellular localization are only now starting to emerge. This is perhaps best illustrated by the polarization of transmembrane hormone (e.g. PINs) and nutrient (e.g. BOR1 and NIP5;1) transporters. Extensive research into the localization of these proteins has clearly demonstrated that plant cells use specific transport and trafficking routes to ensure polar distribution of cargoes to distinct domains. This aspect of polarity implementation has been extensively reviewed, and readers are referred to excellent articles on the topic (Kania et al., 2014; Adamowski and Friml, 2015; Yoshinari and Takano, 2017; Nakamura and Grebe, 2018).

In contrast, the mechanisms that define the specificity of trafficking routes for distinct membrane domains are poorly understood, despite important progress in recent years with the identification of polarly localized regulators of trafficking. Two interesting cases involve the conserved exocyst complex, which functions in polar secretion of PEN3 and NIP5;1 at the outer membrane of epidermal cells (Mao et al., 2016), and in positioning of the Casparian strip in endodermal cells, a process that requires transient polarization by the specialized subunit EXO70A1 independently of secretion (Kalmbach et al., 2017). Another example is the gravistimulation-induced polarization of the LAZY/LAZY-like (LZY)/RCC1-like domain (RLD) module that controls PIN3 polarization during gravitropism, as RLD proteins are putatively involved in a late trafficking step to the membrane via regulation of RAB-E endosomes (Furutani et al., 2020). Notably, the preference of polar cargoes for specific routes can be dynamically modulated during development or environmental adaptation to alter the polarization state. Underlying these dynamics are post-translational modifications of cargo proteins, often by polarly localized regulators (Michniewicz et al., 2007; Kleine-Vehn et al., 2009; Huang et al., 2010; Zhang et al., 2010; Takano et al., 2010), and the activity of condition-specific factors, such as the above-mentioned polar LZY/RLD module (Furutani et al., 2020).

Similar to polar trafficking, we also lack a mechanistic understanding of how other fundamental developmental processes are coordinated by polarity, including the orientation of cell division or the directional intercellular movement of cell fate determinants through plasmodesmata (Perbal et al., 1996; Oikawa and Kyozuka, 2009; Schlereth et al., 2010; Skopelitis et al., 2018; Lu et al., 2018).

## Origin and evolution of plant cell polarity

Our understanding of the mechanisms that give rise to axial polarity in our best-studied model, Arabidopsis, is still full of lacunae, but at least a growing number of polar proteins are known, and some of the details of how their localization is determined are being uncovered. We know next to nothing about what is happening in other members of the Viridiplantae. Thus, it is unclear which aspects of polarity are peculiar to Arabidopsis or its close relatives. Neither is it known which, if any, is deeply conserved components of a core plant polarity toolkit, or how polarity mechanisms changed and elaborated in different lineages during the course of evolution. For instance, filamentous algae belonging to the Zygnematales, the closest sisters to the land plants (embryophytes), grow along a single axis, while the embryophytes themselves grow in three dimensions. How were the mechanisms of axial polarity adapted and elaborated as new polarities, such as inner–outer, were acquired? New cell types and tissues evolved in the descendants of the last common ancestor of land plants, even new organs with new axes, including leaves (Harrison and Morris, 2018), roots (at least twice, independently; Raven and Edwards, 2001; Hetherington and Dolan, 2018), and a complex sporophyte embryo (Radoeva et al., 2019). How were these integrated into the ancestral system of axial polarity, if one existed?

There are indications that at least some features of axial polarity are conserved across land plants. Homologs of the Arabidopsis SOK proteins are found in all land plants for which genome sequence information is available, and in the liverwort *Marchantia* and the moss *Physcomitrium*, SOK orthologs are polarized according to axial cues (van Dop et al., 2020). The single *Marchantia* MpSOK is localized toward the meristematic apical cell, while *Physcomitrium* PpSOK4 is localized toward the inner faces of developing buds, and PpSOK2 to the lower inner edge of bud cells and the proximal medial edge of leaf cells. These findings, together with the fact that orthologs are localized to specific polar domains in distantly related species, points toward the existence of an ancient coordinate system in plant cells (Yoshida et al., 2019; van Dop et al., 2020). Although the DIX domain of SOK proteins is structurally very similar to that of animal DIX and is biochemically equivalent in function to the human domain, suggesting that the involvement of this domain in polarity is exceedingly ancient, the green algal relatives of the land plants lack a bona fide SOK protein. This protein family may have arisen as part of the conquest of the land and the evolution of growth in three dimensions that occurred at this time. Alternatively, DIX domain proteins may have been lost in the algal relatives to land plants.

Canonical PIN proteins, whose highly polarized cellular localization is key to polar auxin flow and thus to many aspects of development in *Arabidopsis*, are present throughout the land plants, while *Klebsormidium*, a charophyte algae, possesses a PIN homolog that functions as a plasma membrane auxin efflux transporter (Bennett, 2015; Skokan et al., 2019). Immunofluorescence detects KfPIN at the periphery of cells, but it does not show clear polarity, and the protein is not polarly localized when expressed heterologously in *Physcomitrium* or Arabidopsis (Skokan et al., 2019). In *Physcomitrium*, the development of the gametophyte begins with a protonemal stage consisting of branching tip-growing filamentous cells. Each filament possesses a single basal–apical axis, and the canonical *Physcomitrium* PINs polarize along this axis, accumulating preferentially toward the apex of the filament. PINs are also expressed once three-dimensional growth begins with the development of leafy gametophores. In the proximal part of the single cell layer thick leaves, PINs are located throughout the plasma membrane but are distributed in an apolar manner, whereas in the distal region they are confined to the proximal and distal ends of the cells (Viaene et al., 2014), demonstrating an organ-wide polarity field of the kind seen in Arabidopsis leaves.

The intrinsic membrane protease DEFECTIVE KERNEL1 (DEK1) also shows a striking cellular polarity during three-dimensional growth in *Physcomitrium*, localizing toward the inner faces of cells in the developing bud where they contact other cells, and absent from the outer domains that face the environment, a pattern seen also in the developing *Physcomitrium* leaf (Perroud et al., 2020). DEK1 may thus be creating or responding to an inner–outer positional cue. Indeed, *dek1* mutants are severely impaired in three-dimensional growth (Perroud et al., 2014). The subcellular polarity of DEK1 in angiosperms has not been determined, but during maize (*Zea mays*), rice, and Arabidopsis embryogenesis, the protoderm fails to be properly specified (Becraft et al., 2002; Johnson et al., 2005; Hibara et al., 2009), hinting at a conserved role for this protein in detecting or responding to the boundary with the exterior early in development.

**Figure 5:**
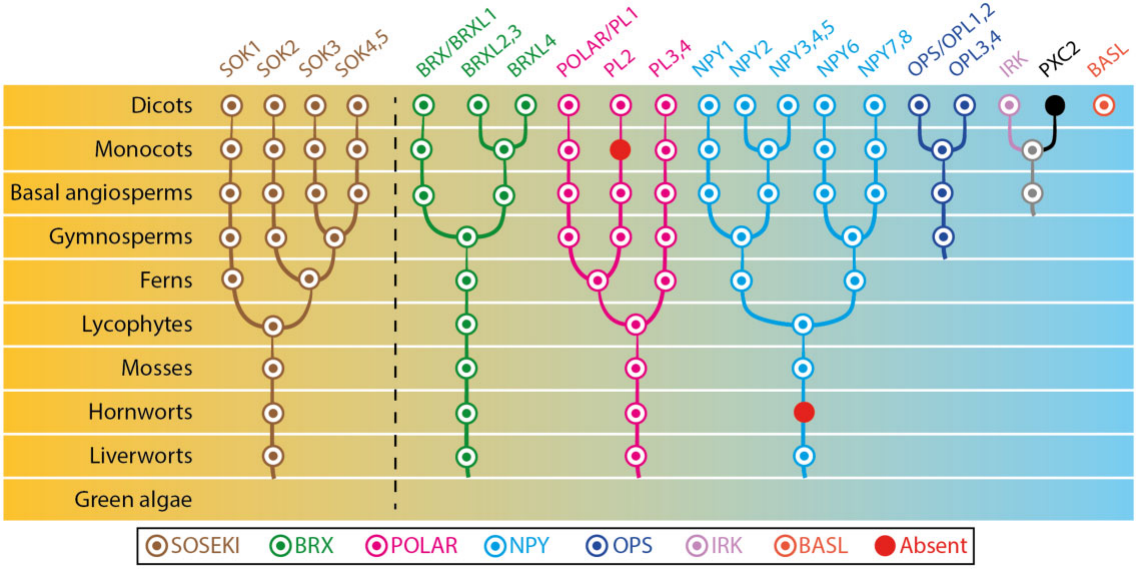
Reconstruction of the ancestral states of polar proteins in plant evolution. Phylogenetic trees show the copy number and phylogenetic relationship of each member in the respective protein families. Each circle is colored according to protein type, as indicated in the box. Names on top correspond to the proteins in *A. thaliana*. Note that only scenarios with strong bootstrap support are shown.

In the light of these tantalizing hints, especially the conserved SOK proteins with their polarization relative to major plant axes, it seems likely that at least some components of axial polarity mechanisms existed in the common ancestor of land plants. Conversely, many polar proteins that have been identified in Arabidopsis are likely to be of restricted phylogenetic distribution. As a first step toward unraveling how the “polarity toolbox” evolved, and distinguishing the core conserved components of the polarity machinery, we have carried out a deep phylogenetic analysis of several well-known polarity regulators to estimate ancestral states across the major lineages during land plant evolution (Mutte et al., 2018; Mutte and Weijers, 2020; Figure 5; Supplemental File S1).

As previously reported, the SOK proteins are deeply conserved, first appearing in the common ancestor of land plants. The bryophyte ancestors possessed a single SOK, although subsequent gene duplications have increased the complement in, for example, the *Physcomitrium* lineage. In the vascular plants, beginning with the ferns, duplications have produced an increasing number of SOK clades.

At the other end of the spectrum lies BASL. The intensive study of this protein in the stomatal lineage has yielded many insights into the mechanisms underlying plant polarity (Dong et al., 2009; Zhang et al., 2015; Mansfield et al., 2018). However, being phylogenetically restricted to the dicots, BASL is not a member of a core polarity mechanism common to land plants. Surprisingly, its partners in stomatal development, POLAR and BRX, are conserved since the common ancestor of land plants. Among the bryophytes, liverworts lack stomata, while these structures are found on the sporophytes of mosses and hornworts (Ligrone et al., 2012). Although the development of moss guard mother cells is regulated by homologs of transcription factors and ligand–receptor pairs that regulate Arabidopsis stomatal development (Caine et al., 2016), the POLAR and BRX homologs have not been studied outside the angiosperms, and it is unknown how deeply conserved their role in stomatal development is. Furthermore, since BRX existed before the appearance of vascular plants, its polar localization and function in vascular cell development were presumably co-opted from some other earlier role. In contrast, OPS, which is localized opposite to BRX at the apical domain of vascular cells (Truernit et al., 2012), first appeared in seed plants, and thus originated in a context in which vascular development already existed.

The LRR-RLK IRK is encoded by a single-copy gene in Arabidopsis that shares a common ancestor with *PXC2* (AT5G01890), with the split occurring at the base of the eudicots. This suggests a specific functional role for IRK in eudicots, which might have resulted from sub-functionalization from the IRK-PXC2 common ancestor. Recent data show that PXC2 shows the same polar localization as IRK (Campos et al., 2020; Goff and Van Norman, 2021), suggesting that the original state of the IRK-PXC2 ancestor was indeed polar. Homologs of IRK are found in all the plant lineages starting with green algae, but IRK belongs to a large family of closely related kinases, and unraveling their evolutionary history will be challenging. Studies in species outside of the eudicots will be necessary to determine whether these homologs polarize and function in ways similar to Arabidopsis IRK.

The NAKED PINS IN YUC MUTANTS (NPY) proteins are members of the NPH3/RPT2-Like (NRL) family of Broad-Complex, Tramtrack, and Bric a brac (BTB) domain-containing proteins that are involved in various developmental processes including phototropism and gravitropism. These proteins likely already existed in the last common ancestor of land plants (previously reported in Suetsugu et al., 2016), although they appear to have been lost in hornworts. There are eight NPY proteins in Arabidopsis, of which NPY1–5 have been characterized and display polar localization very similar to that of PIN proteins, and may be involved in enforcing this polarity (Furutani et al., 2007, 2011; Glanc et al., 2021). Based on our and previous phylogenetic studies (Suetsugu et al., 2016), we named the remaining orthologs as NPY6–8 (Supplemental File S1), although experimental evidence will be required to understand whether these proteins display polar localization and contribute to PIN polarity. The origin of this protein family in the first land plants, where PIN polarization also appears to have first emerged, hints that this interaction may be ancient, although as yet there are no reports of the localization or function of NPY outside of Arabidopsis.

This analysis illustrates that some of the polar proteins that have been heavily studied in Arabidopsis are likely to be relatively recent innovations, while others have deep phylogenetic roots, originating concomitant with and likely facilitating the novelty of 3D growth along multiple axes that appeared with the first embryophytes. They hint at an ancient toolkit of proteins involved in axial polarity that are common to all land plants, although for most of them, it remains to be tested whether they are polarly localized and what functions they might fulfill. Nevertheless, the investigation of axial polarity in lineages outside of Arabidopsis, and especially in the sister lineages of the vascular plants, promises to illuminate the core polarity mechanisms that are shared amongst plants and how new proteins were coopted or innovated to perceive, create, and respond to new polarities and in new contexts as plant development diversified and elaborated during the course of evolution.

## Concluding remarks and future perspectives

Our understanding of cell polarity in plants has grown immensely over the last decade, particularly regarding the molecular mechanisms underlying localized growth and specific aspects of axial polarity implementation. Still, the field is riddled with exciting problems that will surely engage researchers for years to come. We still do not know how axial polarity is established and maintained, how it coordinates downstream events such as cell division plane orientation, or whether it is inherited or re-established after cell division. So far, the regulators of axial polarity remain elusive, and most (if not all) proteins implicated in polarity appear to act as effectors of a pre-established axis. The focus on post-embryonic tissues in most studies of plant polarity might explain this significant gap, if, like in animals (e.g. Kemphues et al., 1988; Perrimon, 1988; Chen et al., 2000; Tao et al., 2009), loss of regulators leads to embryonic lethality. Moreover, symmetry-breaking might only occur in the early embryo and (partially) inherited in subsequent divisions, making the predictable and anatomically simpler early embryo uniquely suited to probe fundamental aspects of polarity. We identify a number of urgent challenges for the field to address:

1. Given that new polar proteins are steadily being identified, it is likely that the polar proteome is substantially complex. Better understanding of polarity will naturally come from expanding the known polar proteome, which will also provide better tools to assess polarity, such as nontransmembrane reporters of domain identity.
2. Although Arabidopsis has been at the forefront of plant polarity research, more emphasis needs to be placed on understanding polarity in a broader phylogenetic sampling of land plants, including bryophytes that lack the anatomic and genetic complexity of vascular and flowering plants. Such studies will also help to reveal the ancient and novel components in plant cell polarity.
3. Clearly, during the initiation of new lateral organs and growth axes, new polarity axes are established. It will be important to identify the nature of the polarity cues or polarity fields that are intrinsic to organs, how such cells reorient their polarity, and how global information is locally interpreted by individual cells.
4. The study of axial plant cell polarity has much to gain from the development and implementation of technological innovations. These include advanced and quantitative image analysis software such as MorphoGraphX (Barbier de Reuille et al., 2015), dedicated polarity quantification tools such as POME (Gong et al., 2021), but also next-generation protein interaction mapping approaches, such as proximity ligation (Branon et al., 2018).

## Supplemental data

The following materials are available in the online version of this article.

**Supplemental File S1.** Methods for phylogenetic analysis of polar proteins.

## Supplementary Material

koab203_Supplementary_Data

## Notes

Conceptualization: V.J, J.J.R., D.W.; Methodology: S.K.M.; Data Curation: S.K.M.; Writing – Original Draft: V.J., J.J.R. D.W.; Writing – Review and Editing: V.J., J.J.R., D.W.; Visualization: V.J, S.K.M., J.J.R; Supervision: D.W.; Project Administration: D.W.; Funding Acquisition: D.W.

The author responsible for distribution of materials integral to the findings presented in this article in accordance with the policy described in the Instructions for Authors (https://academic.oup.com/plcell) is: Dolf Weijers (dolf.weijers@wur.nl)
