# Supplementary material for: Pole position: How plant cells polarize along the axes
Source: Plant Cell. 2021 Aug 2;34(1):174–92. doi: 10.1093/plcell/koab203 (PMC8774072; doi:10.1093/plcell/koab203)
Supplement: koab203_Supplementary_Data [file koab203_supplementary_data.pdf]

Supplemental File S1

To understand the evolutionary patterns and infer the ancestral states of these protein families across all the major land plant lineages we have deployed a pipeline that was developed earlier (Mutte et al., 2018; Mutte and Weijers, 2020). In brief, we used respective protein sequences in each family from *Arabidopsis thaliana*, as query sequences to search against the One thousand plant transcriptomes (OneKP) dataset (One Thousand Plant Transcriptomes Initiative, 2019; Carpenter et al., 2019). Where applicable, we also used homologs from *Marchantia polymorpha* and *Physcomitrium patens* as queries to enhance the scope of homolog detection. The transcripts with high confidence were used for reciprocal BLAST against whole *A. thaliana* proteome to infer the bi-directional orthologous relationship. Sequences for the phylogeny construction were aligned using 'genafpair' algorithm in MAFFT (v7) (Katoh and Standley, 2013). Depending on the alignment quality, positions with 20-30% gaps were removed before the phylogeny construction using TrimAl (Capella-Gutiérrez et al., 2009). Finally, phylogenetic trees were created using Maximum Likelihood (ML) method implemented in IQ-tree with 1000 rapid bootstraps (v2.0.4) (Minh et al., 2020). All the trees were built with JTT as the model of evolution as predicted by the ModelFinder executed through IQtree (Kalyaanamoorthy et al., 2017). Phylogenetic trees were visualized, annotated and shared using iTOL (https://itol.embl.de/shared/dolfweijers). Based on phylogenetic data we have named AT5G47800, AT3G26490, and AT1G67900 as NPY6, NPY7, and NPY8, respectively.

Supplemental References

Capella-Gutiérrez, S., Silla-Martínez, J.M., and Gabaldón, T. (2009). trimAl: a tool for automated alignment trimming in large-scale phylogenetic analyses. Bioinformatics 25: 1972–1973.

Carpenter, E.J. et al. (2019). Access to RNA-sequencing data from 1,173 plant species: The 1000 Plant transcriptomes initiative (1KP). Gigascience 8.

Kalyaanamoorthy, S., Minh, B.Q., Wong, T.K.F., von Haeseler, A., and Jermiin, L.S. (2017). ModelFinder: fast model selection for accurate phylogenetic estimates. Nature Methods 14: 587–589.

Katoh, K. and Standley, D.M. (2013). MAFFT multiple sequence alignment software version 7: improvements in performance and usability. Mol Biol Evol 30: 772–780.

Minh, B.Q., Schmidt, H.A., Chernomor, O., Schrempf, D., Woodhams, M.D., von Haeseler, A., and Lanfear, R. (2020). IQ-TREE 2: New Models and Efficient Methods for Phylogenetic Inference in the Genomic Era. Mol Biol Evol 37: 1530–1534.

Mutte, S.K., Kato, H., Rothfels, C., Melkonian, M., Wong, G.K.-S., and Weijers, D. (2018). Origin and evolution of the nuclear auxin response system. eLife 7: e33399.

Mutte, S.K. and Weijers, D. (2020). High-resolution and Deep Phylogenetic Reconstruction of Ancestral States from Large Transcriptomic Data Sets. Bio Protoc 10.

One Thousand Plant Transcriptomes Initiative (2019). One thousand plant transcriptomes and the phylogenomics of green plants. Nature 574: 679–685.
